# Tie2 as a novel key factor of microangiopathy in systemic sclerosis

**DOI:** 10.1186/s13075-017-1304-2

**Published:** 2017-05-25

**Authors:** Falk Moritz, Janine Schniering, Jörg H. W. Distler, Renate E. Gay, Steffen Gay, Oliver Distler, Britta Maurer

**Affiliations:** 1 Department of Rheumatology, University Hospital Zurich, Gloriastrasse 25, 8091 Zurich, Switzerland; 2 Department of Oncology, St. Georg Hospital, Leipzig, Germany; 3 Department of Internal Medicine 3, University Hospital, Erlangen, Germany

**Keywords:** Microvasculopathy, Systemic sclerosis, Angiopoietins, Tie2

## Abstract

**Background:**

The angiopoietin(Ang)/Tie2 system is a key regulator of vascular biology. The expression of membrane bound (mb) Tie2 and Ang-1 ensures vessel stability, whereas Ang-2, inducible by vascular endothelial growth factor (VEGF), hypoxia, and inflammation, acts as an antagonist. Tie2 signalling is also attenuated by soluble Tie2 (sTie2), the extracellular domain of the receptor, which is shed upon stimulation with VEGF. Herein, we investigate the role of Ang/Tie2 in the peripheral vasculopathy in systemic sclerosis (SSc) including animal models.

**Methods:**

The expression of Ang-1/-2 and Tie2 in skin/serum of SSc patients was compared with healthy controls by immunohistochemistry (IHC)/ELISA. Expression of Ang/Tie2 was analysed in different animal models: VEGF transgenic (tg) mice, hypoxia model, bleomycin-induced skin fibrosis, and tight skin 1 (TSK1) mice.

**Results:**

In SSc, dermal microvessels abundantly expressed Ang-2, but not Ang-1 compared with healthy controls. The percentage of mbTie2+ microvessels was profoundly decreased whereas the levels of sTie2 were increased already in early disease. Both in skin and sera of SSc patients, the Ang1/2 ratio was reduced, being lowest in patients with digital ulcers indicating vessel destabilizing conditions. We next studied potential influencing factors in animal models. The VEGF tg mouse model, the hypoxia, and the inflammation-dependent bleomycin model all showed a similar dysregulation of Ang/Tie2 as in SSc, which did not apply for the non-inflammatory TSK1 model.

**Conclusion:**

Peripheral microvasculopathy in SSc results from a complex dysregulation of angiogenic signalling networks including the VEGF and the Ang/Tie2 system. The profoundly disturbed Ang-/Tie-2 balance might represent an important target for vascular therapeutic approaches in SSc.

**Electronic supplementary material:**

The online version of this article (doi:10.1186/s13075-017-1304-2) contains supplementary material, which is available to authorized users.

## Background

Microvascular injury due to dysregulation of angiogenic factors such as vascular endothelial growth factor (VEGF) and hypoxia [1–4] is a very early pathogenic event in systemic sclerosis (SSc) [5]. The Angiopoietin(Ang)/Tie2 system is a key regulator of angiogenesis. The binding of Ang-1, produced by vascular smooth muscle cells and other perivascular cells, to the membrane-bound (mb) Tie2 receptor on endothelial cells (EC) is crucial for vessel stability. Tie2 activation promotes vessel assembly and maturation by mediating survival signals for EC and regulating the recruitment of pericytes [6]. Ang-2, inducible in EC by VEGF, hypoxia, and inflammation [6–8], antagonizes the Ang-1/Tie2 pathway by competing with Ang-1. Both ligands bind to Tie2 with similar affinity. Under physiologic conditions, the levels of Ang-1 exceed those of Ang-2, thereby ensuring the quiescent state of the vasculature. Increased levels of Ang-2 are associated with vascular remodelling [9]. Furthermore, the effects of Ang-2 are VEGF-dependent. In the absence of VEGF, Ang-2 has vessel destabilizing effects and induces regression of microvessels [10, 11]. In the presence of VEGF, Ang-2 induces angiogenesis with endothelial cell proliferation, migration and disruption of the vascular basement membrane due to the increased expression of matrix metalloproteinases [12]. Thus, the Ang-1/-2 ratio determines the functional status of the local vasculature. In contrast to mbTie2, little is known about soluble Tie2 (sTie2), the extracellular domain of the Tie2 receptor, which is released by proteolytic cleavage by matrix metalloproteases upon stimulation with VEGF in a process also referred to as shedding [13, 14]. sTie2 is detectable in healthy individuals, and increased serum concentrations were measured in cardiovascular diseases, diabetic retinopathy [15], and SSc [16, 17].

**Fig. 1:**
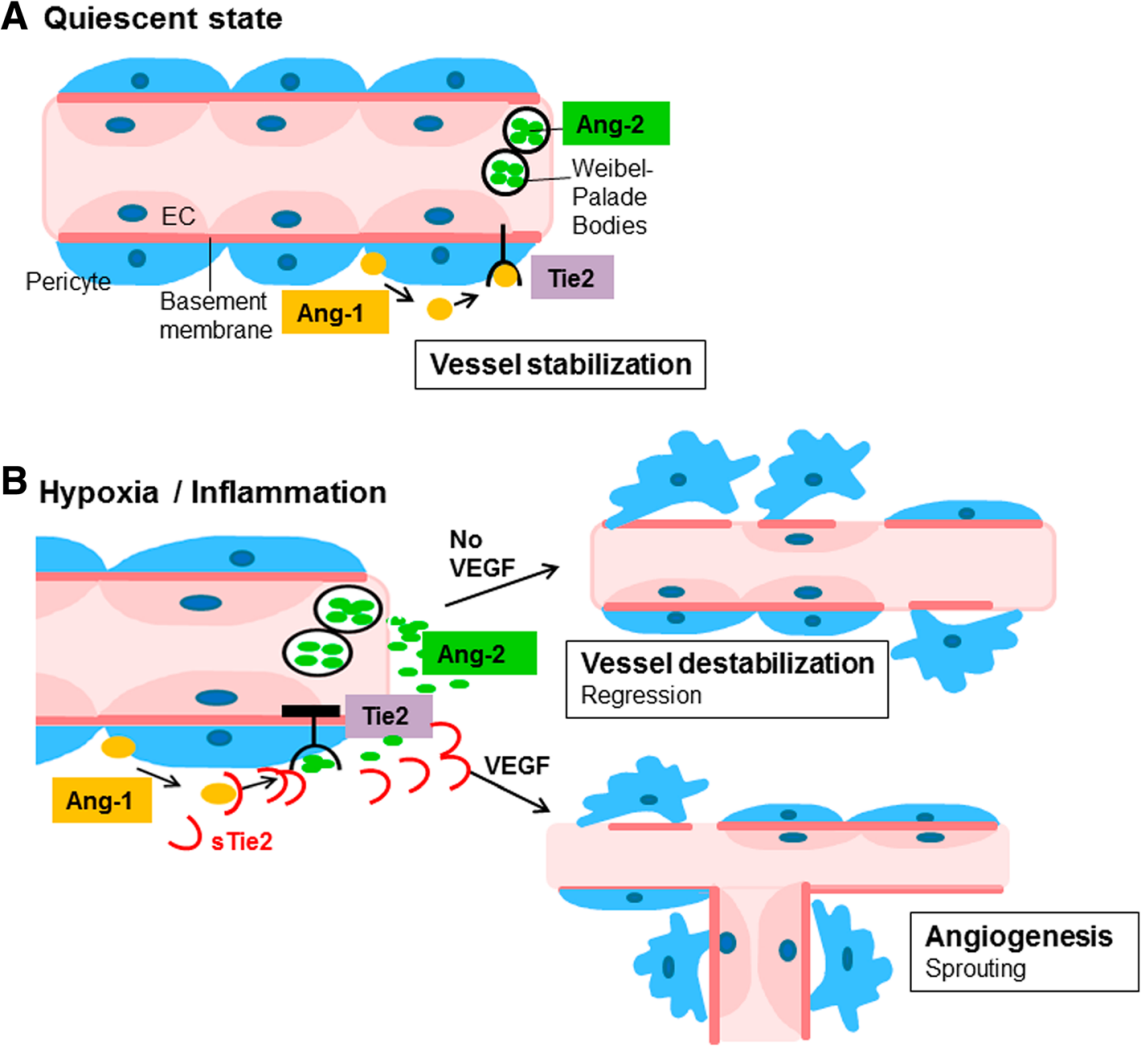
The role of Angiopoietins and Tie2 in vascular biology (adapted from [6–8, 11, 13, 14]). **a** In quiescent state, Ang-1, produced by pericytes, acts in a paracrine manner on the membrane-bound (mb) Tie2 receptor on endothelial cells (*EC*). The effects of Ang-1/Tie2 signalling ensure vessel stabilization. Ang-2, produced by EC, is stored in Weibel-Palade bodies. **b** In hypoxia and inflammation, Ang-2 is released from the Weibel-Palade bodies and acts in an autocrine manner on EC. Due to competitive binding to mbTie2, Ang-2 antagonizes Ang-1 signalling. In the absence of VEGF, the inhibition of Tie2 signalling leads to vessel destabilization and regression due to apoptosis of EC, loss of pericyte coverage and disrupture of the basement membrane. In the presence of VEGF, Ang-2 exerts pro-angiogenic effects with proliferation and migration of EC and sprouting of new branches. Additionally, VEGF causes shedding of Tie2. sTie acts as competitive ligand for Ang-1/-2 thereby blocking downstream signalling with anti-angiogenic effects. *Ang* Angiopoietin, *VEGF* vascular endothelial growth factor

Previous studies supported an equal affinity of Ang-1 and Ang-2 for the Tie2 receptor [10, 11, 13]. Interestingly, a recent study reported a higher affinity of Ang-1 to sTie2, which indicates that sTie2 might shift the Ang-1/Ang-2 ratio in favour of Ang-2 thereby inducing destabilization and regression of microvessels [18]. Other studies suggest competitive binding to mbTie2 thereby blocking access for Ang-1/-2 with inhibition of downstream signalling [6]. The scheme in Fig. 1 summarizes the current concept of the role of Ang/Tie2 in vascular remodelling.

In the present study, we assessed alterations of the Ang/Tie2 system in SSc-associated dermal microvasculopathy and in different animal models of SSc.

## Methods

### Patients

Serum samples were obtained from patients with limited cutaneous (lc) SSc (n = 21) and diffuse cutaneous (dc) SSc patients (n = 13) fulfilling the LeRoy criteria [19], patients with early SSc not yet fulfilling the LeRoy criteria (= pre-SSc) (n = 12), and from age- and sex-matched healthy donors (n = 40). The patients were 56.2 ± 11.6 years old, the majority was female (75%). The average disease duration was 6.3 ± 4.64 years. All patients had Raynaud’s phenomenon, 68% had a history of digital ulcers, 37% had current digital ulcers, and 93% had teleangiectasias. The majority of patients were ANA positive (88.3%). A total of 39% were positive for anti-Scl-70, 44% for anti-centromere, and 2% for anti-RNA-polymerase III. The majority of patients had an active pattern in the nailfold capillaroscopy, whereas 33% showed a late, and 17% an early, pattern [20]. Skin biopsies were obtained by punch biopsies from involved skin at the forearms from 24 of the above mentioned SSc patients (13 lcSSc, 11 dcSSc) and from 19 of the above mentioned healthy controls. All participants had signed a consent form approved by the local institutional review boards. The study had been approved by the cantonal ethics committee Zurich.

### Animals

#### VEGF tg mice

To investigate the effect of VEGF on Ang/Tie2, VEGF homo- (+/+) (*n* = 8) and heterozygous (+/−) (*n* = 9) transgenic (tg) mice and wildtype (wt) littermates (*n* = 6/9) were analysed. VEGF tg mice have been described previously [21]. Briefly, in VEGF tg mice, the murine VEGF164 gene which is the murine equivalent to VEGF-A165, was cloned into a human keratin 14 promoter expression cassette which had previously been shown to selectively target transgene expression to basal keratinocytes of the skin [21]. VEGF tg mice were on the FVB background.

#### Hypoxia model

Four- to six-week-old female C57BL/6 mice (n = 3) were exposed to systemic normobaric hypoxia by substitution of oxygen with nitrogen in a closed Perspex chamber using a Digamix 2 M 302/a-F pump (H. Woesthoff GmbH, Bochum, Germany) at a flow rate of 37 l/min. When mice were placed into the chamber, O_2_ fractions were decreased gradually from 21 to 6% within 1 h and mice were kept in the hypoxic chamber for additional 24 to 48 h. In parallel, nine littermates were kept under normoxic conditions. The experimental set-up was described previously [4].

#### Bleomycin-induced skin fibrosis in mice

The bleomycin model is a frequently used model of chemically induced dermal fibrosis. The bleomycin-induced skin fibrosis in mice is considered as an animal model that mimics early, pro-inflammatory stages of skin fibrosis in SSc. Skin fibrosis was induced in 6- to 8-week-old C57/BL6 mice (n = 4) by local injections of bleomycin for 21 days as previously described [22]. Briefly, 100 μl of bleomycin dissolved in 0.9% sodium chloride (NaCl) at a concentration of 0.5 mg/ml was administered every other day by subcutaneous injections in defined areas of the upper back. Subcutaneous injections of 100 μl 0.9% NaCl were used in a second group (n = 4) as controls.

#### TSK1 mice

Compared to the bleomycin model, tight skin 1 (TSK1) mice [23] are considered as a late-stage, non-inflammatory model of skin fibrosis in SSc. Due to a dominant mutation of the fibrillin 1 gene, TSK1 mice develop an increased dermal and especially hypodermal thickness. TSK1 mice were interbred with pa/pa mice in which a recessive mutation (pa) induces a light grey colour of the fur and pink eyes. Because the fibrillin 1 gene is genetically linked to the pa gene, mice can be screened for the tsk1 mutation based on the colour of eyes and fur. All mice with black fur and eyes carry the dominant tsk1 mutation and are heterozygous for the pa mutation. In contrast, mice with grey fur and pink eyes do not carry the tsk1 mutation, but are homozygous for the mutated pa gene. Apart from changing the skin colour, the pa mutation does not alter skin physiology and has no impact on skin fibrosis [23]. In the current experiments, four TSK1 and four pa/pa mice were analysed.

The animal protocols were approved by the cantonal veterinary office Zurich.

### Immunohistochemistry

Murine and human skin samples were fixed in 4% formalin and embedded into paraffin as described previously [24]. For immunohistochemistry, 5 μm thick sections of human and murine skin were used. Immunohistochemical double stainings were performed in sequential order. On the first day, after pre-treatment with citrate buffer, the slides were incubated with primary rabbit polyclonal von Willebrand Factor (vWF) antibody (Abcam, Cambridge, UK) at room temperature for 1 h, followed by incubation with biotin-labelled secondary goat anti-rabbit antibody (Jackson ImmunoResearch, Soham, UK). Staining was visualized with ABC phosphatase kit (Vector Laboratories, Burlingame, CA, USA) and developed using Vector blue. After additional heat-mediated treatment with citrate buffer, sections were incubated with primary rabbit polyclonal Ang-1 or Ang-2 antibodies (Abcam) overnight at 4 °C, followed by incubation with biotin-labelled secondary goat anti-rabbit antibodies (Jackson ImmunoResearch). Stainings were visualized using an ABC peroxidase kit and developed using DAB (Vector Laboratories). The sequential staining with Tie2 was performed similarly with the exception that no additional heat-mediated removal was required on the second day since the primary Tie2 antibody was mouse monoclonal (Abcam). Isotype-matched IgGs were used as negative controls.

### Analysis of skin sections

The analyses of all immunohistochemical stainings were performed by two independent examiners who were blinded with regard to the different groups. All slides were analysed twice by each examiner. In case of a variation of the results >10%, the respective slides were re-assessed to reach consensus. Pictures were taken with a slide scanner (Zeiss Axio Scan.Z1), using Zen lite software (blue edition 2.3) (Carl Zeiss Microscopy GmbH, Jena, Germany).

For the semi-quantitative analysis of microvessel density, pictures of five randomly chosen high power fields (HPF)/slide were taken. Then, vWF+ blood vessels (dermal microvessels) were counted. Either the ratio or the percentage of Ang-1+, Ang-2+, or Tie2+ blood vessels was determined by assessing their numbers referred to the total number of vWF+ microvessels/HPF using the respective double stainings. Blood vessel density was assessed in the dermis without the epidermal and the subdermal layers, except for the TSK1 mice in which the hypodermis was analysed. The number/percentage of microvessels was then calculated for each group using GraphPad Prism software (GraphPad Software Inc., San Diego, CA, USA).

### ELISA

Serum concentrations of Ang-1/-2 and sTie2 were measured using the colorimetric sandwich ELISA technique (Quantikine; R&D Systems, Abingdon, UK).

### Real-time PCR

Biopsy specimens (0.5 cm2) were homogenized with TissueLyser (Qiagen, Basel, Switzerland). Total RNA was isolated using the RNeasy Mini Kit (Qiagen, Hombrechtikon, Switzerland) and reverse transcribed into complementary DNA (cDNA) with random hexamers [25]. Expression of murine Ang-1, Ang-2, TEK (= Tie), and 18S rNA was measured using TaqMan Gene Expression Assays (Applied Biosystems, Basel, Switzerland). qPCR was performed using a 7500 Real-Time PCR System (Applied Biosystems). All qPCR experiments were performed in duplicate.

### Cell culture, reagents, stimulation assay, and analysis

Human dermal microvascular endothelial cells (HMVEC-d adult, Cambrex, Rockland, ME, USA) were cultured in six-well plates in endothelial cell medium (EGM-2 MV, Lonza, Basel, Switzerland), and passages 3–8 were used for analysis. After 24 h of serum reduction, HMVECs were stimulated for 12, 24, and 48 h with recombinant VEGF-A165 (R&D Systems) at concentrations of 2.5, 5, and 10 ng/ml. Controls were exposed to equivalent dosages of the carrier protein BSA [26]. sTie was measured by ELISA as described above in cell culture supernatants. The percentage of Tie2+ cells was evaluated using flow cytometry analysis (FACS) according to the instructions of the manufacturer. Briefly, HMVECs were washed with PBS, detached with trypsin/0.5% EDTA and centrifuged at 200 × g for 5 min. Afterwards cell pellets were resuspended and incubated for 15 min at room temperature with the primary Tie2 antibody. Isotype-matched IgGs were used as negative controls. The analysis was performed using the FACScan flow cytometer (Becton Dickinson, Mansfield, MA, USA). Data were analysed with CellQuest software (Becton Dickinson Immunocytometry Systems, San Jose, CA, USA).

### Statistical analysis

For statistical analysis, GraphPad Prism software (version 5.01) was employed (GraphPad Software Inc.). Normal distribution of data was examined using the Kolmogorov-Smirnov test. For parametric non-related data, expressed as mean ± standard error of the mean (SEM), the unpaired two-tailed *t* test was used. Nonparametric non-related data, expressed as median_(Q1, Q3)_ were analysed employing the Mann-Whitney *U* test. *P* values less than 0.05 were considered statistically significant.

Information on the histology and assessment of skin fibrosis in the bleomycin model and the measurement of collagen contents by hydroxyproline assay is provided in Additional file 1 supplemental methods.

## Results

### Dysregulation of the Ang/Tie2 system in the dermal microvasculopathy in SSc

**Fig. 2:**
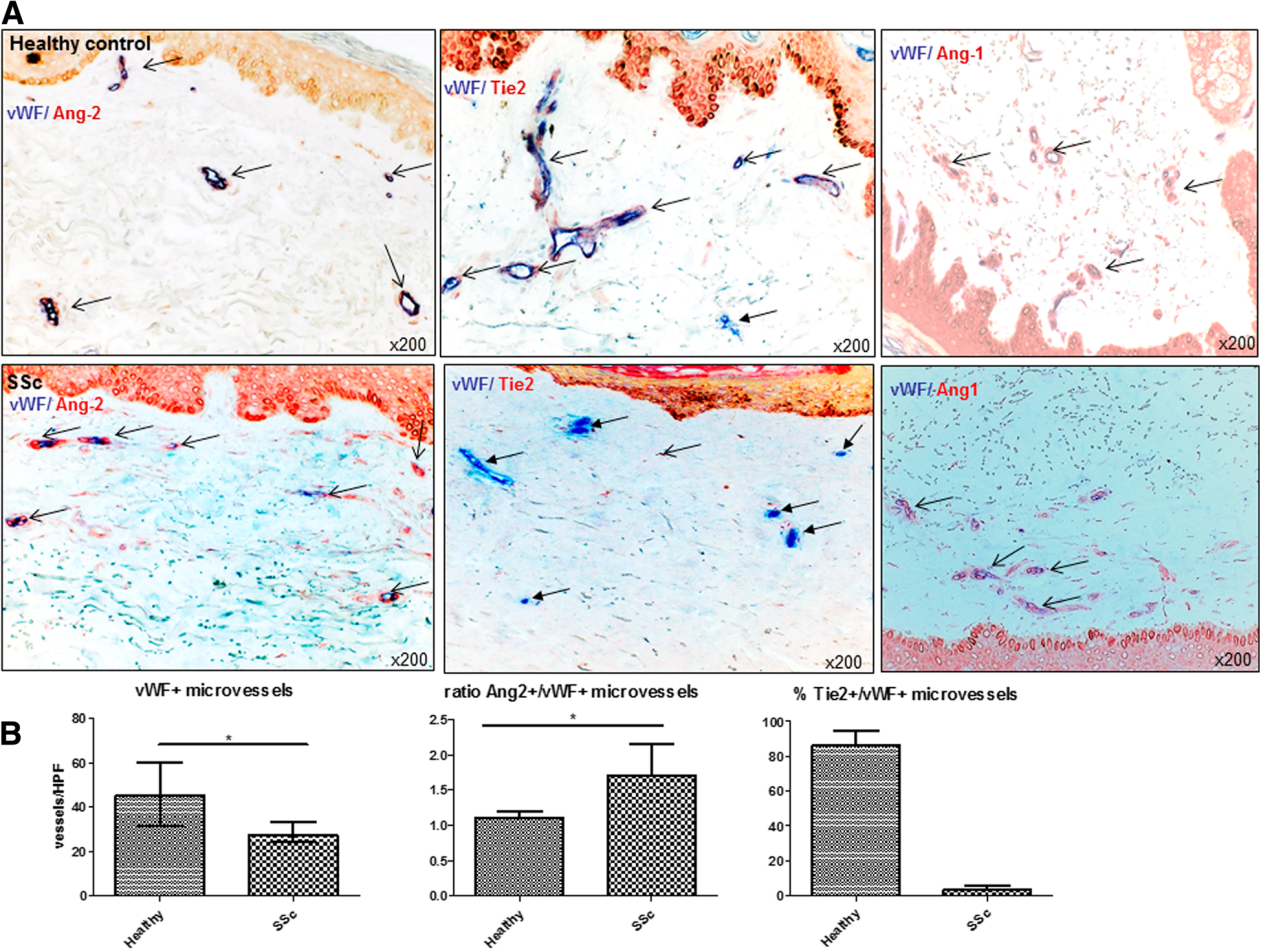
Expression of Ang/Tie2 in dermal microvessels of SSc patients and controls. **a** As assessed by double (+/+) staining of skin biopsies, Ang-2 (*red* staining) was more abundantly expressed in dermal microvessels (vWF+; *blue* staining) of SSc patients compared with healthy controls, whereas Ang-1 (*red* staining) did not differ. The expression of the membrane-bound Tie2 receptor (*red* staining) was remarkably reduced in dermal microvessels (vWF+; *blue* staining) of SSc patients compared with healthy controls. Vessels are indicated by *arrows*. **b** shows the respective semi-quantitative analyses of dermal Ang-2 and mbTie2 expression as well as the reduced microvascular density of SSc patients compared with healthy controls. Pictures are representative examples of 24 SSc patients and 19 healthy controls. *Ang* Angiopoietin, *SSc* systemic sclerosis, *vWF* von Willebrand factor

**Fig. 3:**
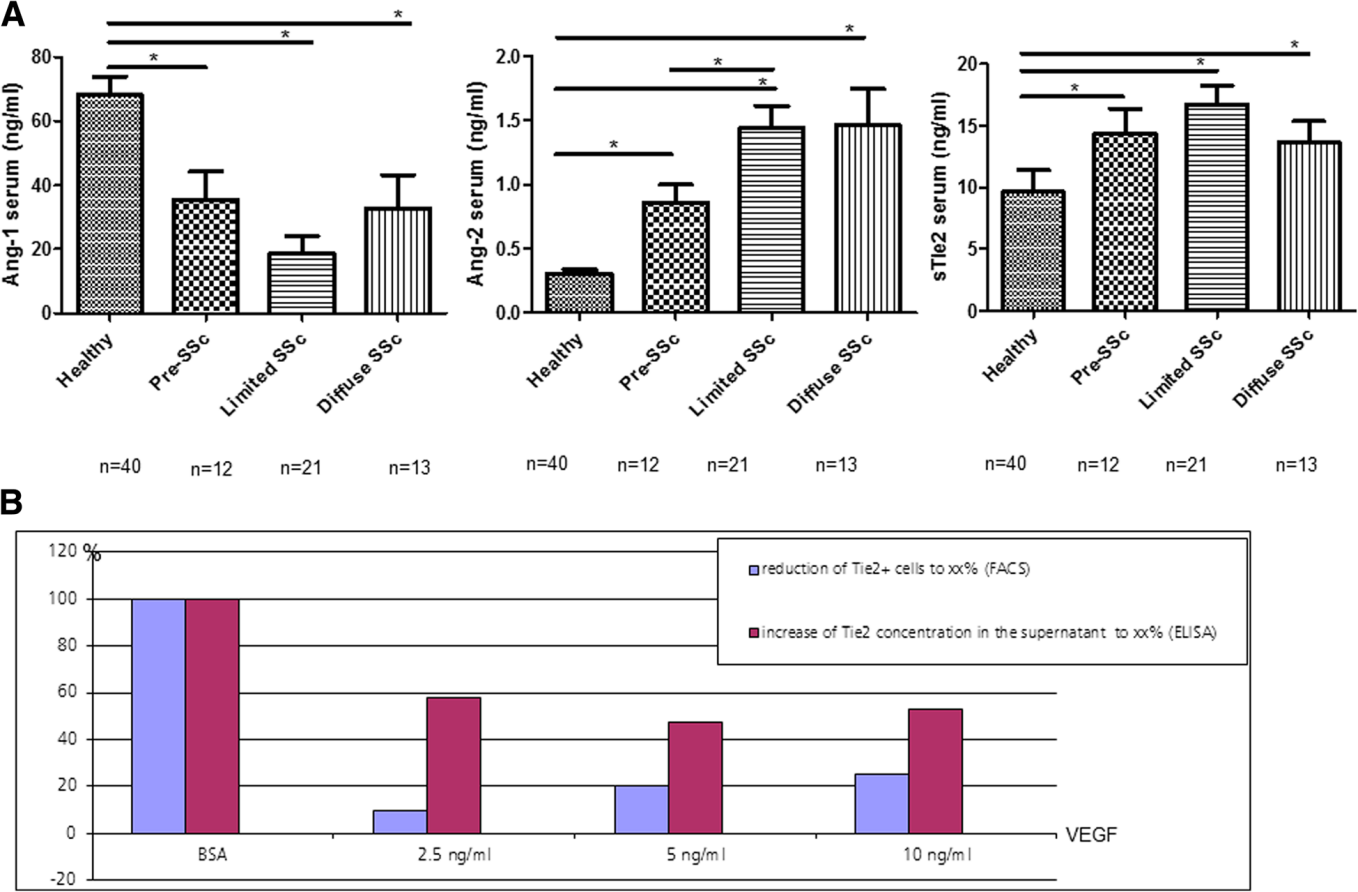
Serum levels of Ang/Tie2 in SSc patients and controls and effects of VEGF on human dermal microvascular endothelial cells. **a** As assessed by ELISA, serum levels of Ang-2 were significantly increased in patients with pre-SSc as well as in established disease irrespective of the disease subset (lc = limited cutaneous vs. dc = diffuse cutaneous) compared with healthy controls. In contrast, the Ang-1 levels were remarkably decreased in all different SSc subsets compared with healthy controls. Of note, all SSc patients irrespective of cutaneous subtype or disease stage showed elevated serum concentrations of sTie2 compared with healthy controls. **b**. Treatment of dermal HMVECs with recombined human VEGF led to a dose-dependent reduction of Tie2+ cells with concomitant increase of sTie2 in the supernatants thereby supporting the shedding hypothesis. *Ang* Angiopoietin, *SSc* systemic sclerosis, *sTie2* soluble Tie2, *VEGF* vascular endothelial growth factor

In skin biopsies of SSc patients, the microvascular density was generally reduced compared with healthy controls (ratio of vWF+ vessels/HPF 27.3_(24,38)_ vs. 45.5_(31,60)_; *p* = 0.001; Fig. 2a, b). Dermal capillaries of SSc patients expressed Ang-2 more abundantly than healthy controls (Fig. 2a, b) (ratio of Ang-2+/vWF+ microvessels/HPF 1.7_(1.3,2.5)_ vs. 1.1_(0.8,1.3)_; *p* = 0.01). Most impressively, the percentage of mbTie2-positive microvessels was strongly and consistently decreased in the skin of SSc patients compared with healthy controls (median_(quartile range)_ 3.8_(1,6)_% vs. 87_(78,99)_%; *p* < 0.001) (Fig. 2a, b). The expression of Ang-1 did not differ in dermal capillaries of SSc patients compared with healthy controls (Fig. 2a). In the analysed sections, no difference in the expression of Ang-1/-2 or Tie2 could be observed between lcSSc or dcSSc. Furthermore, to evaluate whether the changes on tissue level were reflected on systemic level, we additionally measured the serum levels of Ang-1, Ang-2, and sTie2. To evaluate whether the loss of mbTie2 in dermal microvessels might be due to shedding [13], we assessed the serum levels of sTie2, which is the soluble, cleaved, extracellular domain of the Tie2 receptor. Indeed, sTie2 levels of SSc patients were higher than those of healthy controls (mean ± SEM 9.6 ± 1.7 ng/ml) without differences between the lcSSc or dcSSc subsets (16.7 ± 7.5, 13.6 ± 1.7 ng/ml; *p* < 0.01) (Fig. 3a). In accordance with the shedding hypothesis, treatment of human dermal microvascular endothelial cells (HMVECs) with increasing dosages of VEGF for 12 h reduced the numbers of Tie2-positive cells by 9.6, 20.2, and 25.1% whereas in parallel there was an increase of the sTie2 concentration in the supernatants by 47 to 57% (Fig. 3b). Serum Ang-2 levels were increased in both lcSS and dcSSc patients as compared with healthy controls (1.4 ± 0.2 and 1.5 ± 0.3 vs. 0.3 ± 0.02 ng/ml; *p* < 0.01) whereas the Ang-1 levels where remarkably lower (18.4 ± 5.7 and 32.9 ± 10.3 vs. 68.4 ± 5.4 ng/ml; *p* < 0.01) (Fig. 3a). Irrespective of the cutaneous subtype, in the whole group of SSc patients with established disease, the serum levels of Ang-1 (23.9 ± 5.3), Ang-2 (1.5 ± 0.2), and sTie2 (17.9 ± 1.1) differed significantly from healthy controls (*p* < 0.001, respectively). The imbalance towards Ang-2 in patients with lcSSc and dcSSc resulted in a strongly reduced Ang-1/-2 ratio as compared with healthy controls (13:1 and 23:1 vs. 221:1, respectively; *p* < 0.05. Interestingly, SSc patients with digital ulcers had a significantly lower Ang-1/Ang-2 ratio as compared with those without (2.4_(2,3)_ vs. 7.2_(7,9)_; *p* < 0.02). In contrast, patients with teleangiectasias had a significantly higher Ang-1/Ang-2 ratio (1.9 ± 0.4 vs. 1.3 ± 0.08; *p* < 0.05) than those without. Most importantly, in patients with pre-SSc, serum levels of Ang-1/-2 already differed significantly from healthy controls (35.5 ± 8.8 vs. 68.4 ± 5.4 ng/ml and 0.9 ± 0.2 vs. 0.3 ± 0.02 ng/ml; *p* < 0.02) (Fig. 3a). Serum levels of sTie2 were as high as in patients with established disease and thus remarkably elevated compared with healthy controls (14.4 ± 1.9 vs. 9.6 ± 1.8 ng/ml; *p* = 0.01) (Fig. 3a). Of note, in line with previous results [2, 3], pre-SSc patients also had significantly elevated serum VEGF levels compared with healthy controls (481_(120,559)_ vs. 101_(10,169)_; *p* = 0.01), which were as high as in patients with established disease (lcSSc 412_(250,609)_ and dcSSc 634_(404,941)_ pg/ml).

In summary, we observed a severe disturbance in the expression of Ang-1 and Ang-2 as well as of Tie2 in the skin and sera of patients SSc.

### Potential influences on the Ang/Tie2 expression in vivo

To assess which pathogenic factors in SSc might affect the expression of Ang/Tie2, we evaluated pathophysiologically different animal models of SSc. First, we studied the Ang/Tie2 system in a vascular model of SSc, using the VEGF tg mice since VEGF plays a key role in SSc-associated microvasculopathy and has been suggested to strongly influence the effects of Ang/Tie2 on vascular remodelling [6, 13] (Fig. 1). As previously shown, VEGF tg mice develop a proliferative vasculopathy with tortuous and dilated capillaries with increased vessel wall thickness reminiscent of both the capillary changes observed in SSc [1] and in Ang-2-overexpressing mice [11]. In VEGF+/+ tg mice as compared with VEGF+/- tg mice, chronically high levels of VEGF dampen the pro-angiogenic effects of VEGF [1], which is similarly observed in SSc patients with a chronically elevated VEGF levels and insufficient angiogenesis [2, 3].

**Fig. 4:**
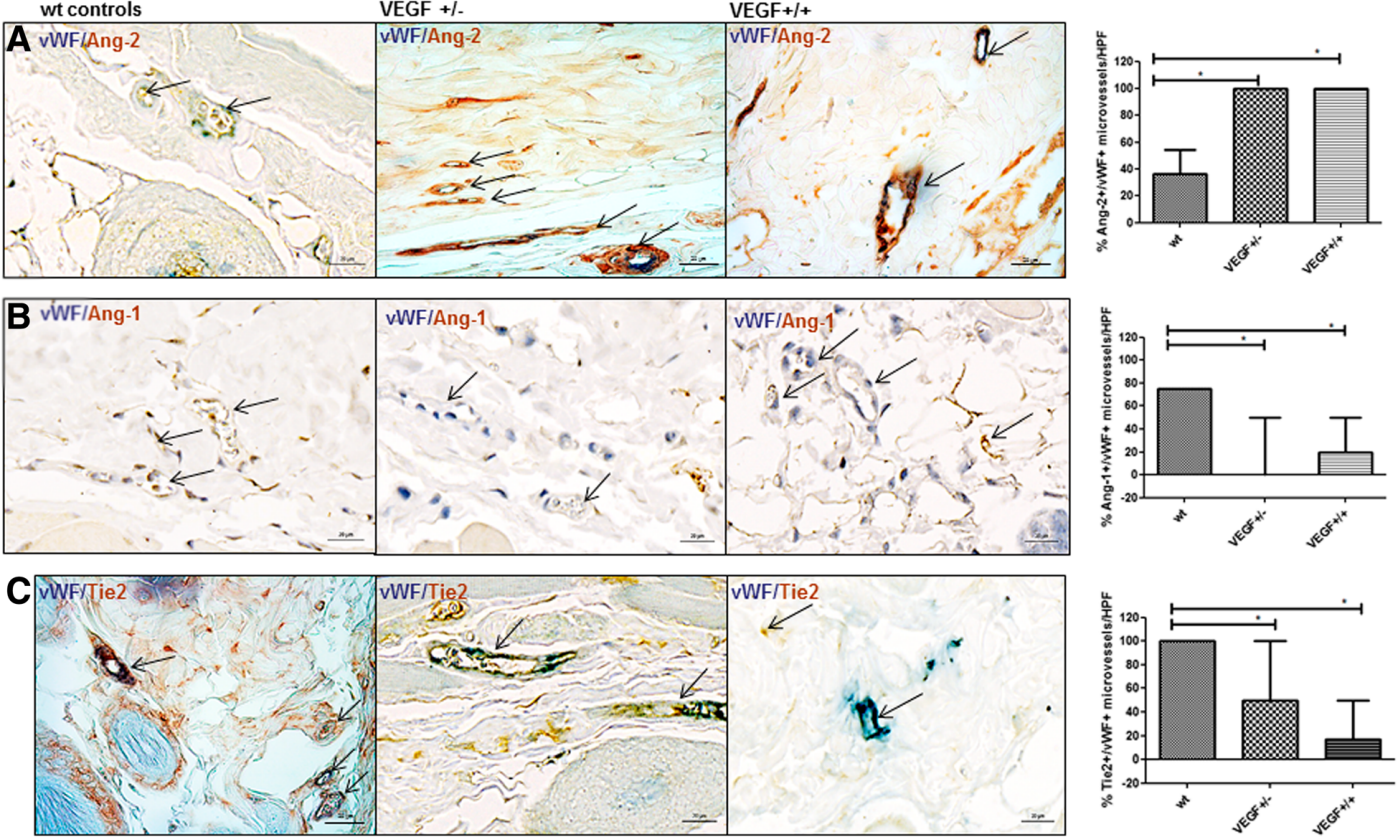
Expression of Ang/Tie2 in dermal microvessels of VEGF tg mice. Overall, the changes in VEGF tg mice reflected the changes observed in human SSc. **a** In VEGF +/- tg mice, the percentage of Ang-2 positive (*brown* staining) dermal microvessels (vWF positive, *blue* staining) was significantly increased compared with wt mice (vessels indicated by *arrows*). The same could be observed in VEGF +/+ tg mice. **b** In contrast, VEGF +/- and VEGF+/+ tg mice showed a lower percentage of Ang-1 (*brown* staining) dermal microvessels (vWF positive; *blue* staining) compared with controls. Double-stained vessels are indicated by *arrows* with an open *arrowhead* whereas vessels being only positive for vWF are indicated by *arrows* with a closed *arrowhead*. **c** The expression of membrane-bound Tie2 (mbTie) (*brown* staining) was remarkably reduced in the microvessels (vWF positive; *blue* staining) of VEGF +/- and +/+ tg mice compared with wt mice Vessels are indicated *by arrows*. Pictures are representative examples of +/-- (+/−) (*n* = 9) and +/+ (+/+) (*n* = 8) VEGF tg mice and respective controls (*n* = 6/9). *+/-* heterozygous, *+/+* homozygous, *Ang* Angiopoietin, *vWF* von Willebrand factor, *wt* wildtype

In VEGF-+/- and VEGF+/+ tg mice, a higher proportion of dermal microvessels (vWF+) expressed Ang-2 compared with wt mice (% of Ang-2+/vWF+ microvessels/HPF 100_(78,100)_ and 100_(100,100)_ vs. 36.7_(0,57)_; *p* = 0.004, *p* = 0.0003) (Fig. 4a), whereas Ang-1 was reduced (% of Ang-1+/vWF+ microvessels/HPF 0_(0,50)_ and 20_(0,50)_ vs. 75_(66.7,75)_; *p* = 0.04, *p* = 0.05) (Fig. 4b). Of note, expression of mbTie2 was remarkably reduced in the dermal capillaries (vWF+) of both VEGF+/- and VEGF+/+ tg mice as compared with controls (% of Tie2+/vWF+ microvessels/HPF 50_(25,100)_ and 16.7_(0,50)_ vs. 100_(88.3,100)_; *p* = 0.03, *p* = 0.0006) (Fig. 4c). These observations are in line with previous data showing that VEGF tips the balance towards Ang-2 and decreases the local expression of mbTie2 [27]. Furthermore, the decreased Ang-1/-2 ratio with a strong concomitant loss of mbTie2 in chronic VEGF exposure in vivo mirrored the observed changes in SSc (Fig. 2).

Tissue hypoxia is a known phenomenon in SSc [2, 4]. To evaluate whether hypoxia might contribute to the dysbalance of the Ang/Tie2 system in SSc, we analysed the tissue expression in hypoxic compared to normoxic mice. Whereas the expression of Ang-1 mRNA did not change, Ang-2 and Tie2 mRNA transcripts increased on the tissue level in hypoxia (2.4 ± 0.4-fold; *p* = 0.027 and 2.6 ± 0.8-fold; *p* = 0.042) compared to normoxia (Additional file 2). Our data are in accordance with previous studies showing that hypoxia increases the expression of Ang-2 [6]. Since Ang-2 blocks Tie2 signalling, this ultimately leads to vessel destabilization and regression [6].

**Fig. 5:**
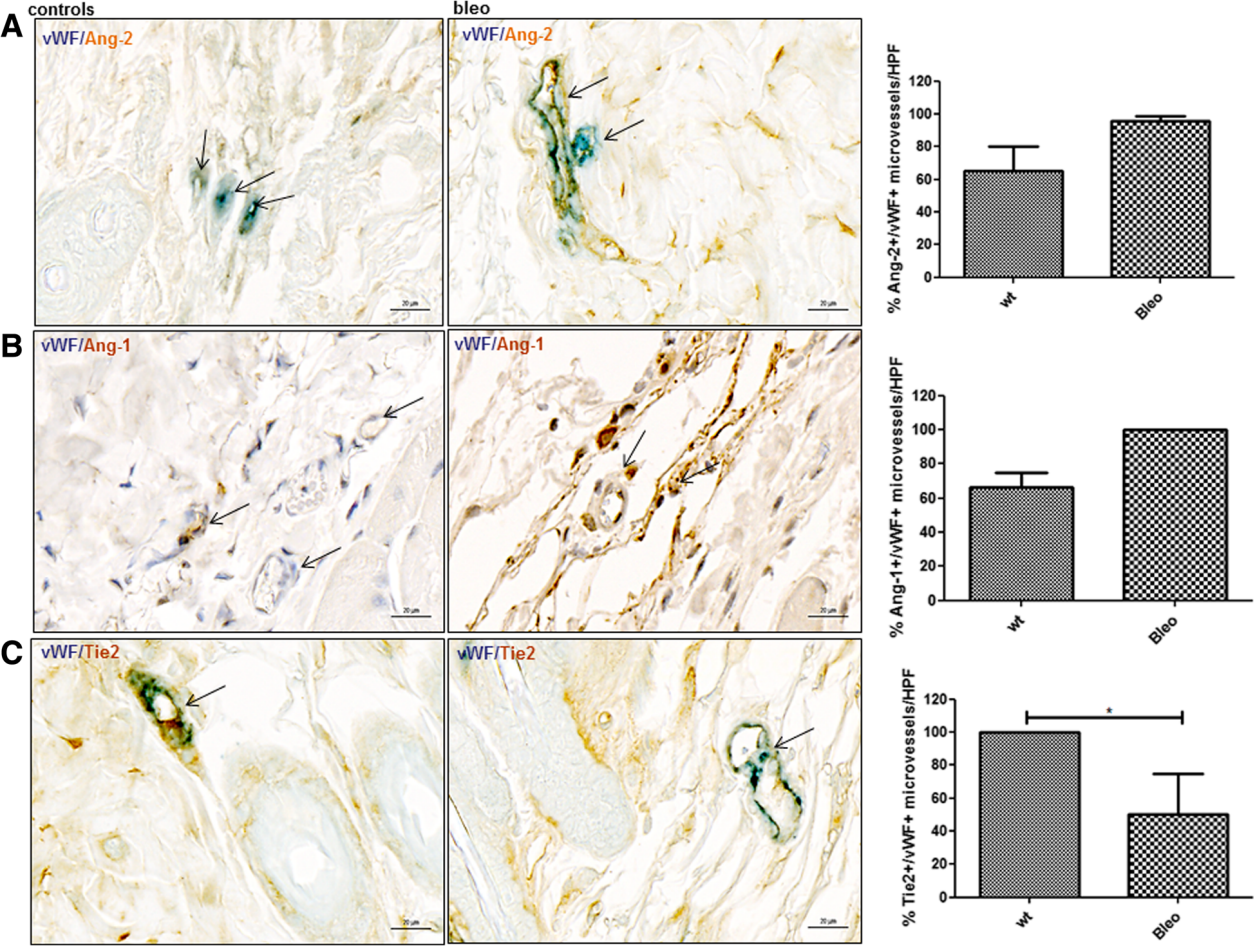
Expression of Ang/Tie2 in dermal microvessels of bleomycin-challenged mice. **a**, **b** In bleomycin-treated mice, no significant difference in the expression of Ang-2 or Ang-1 (*brown* staining) could be observed in dermal microvessels (vWF positive; *blue* staining) compared with saline-treated controls. Vessels are indicated by *arrows*. **c** The percentage of mbTie2 positive (*brown* staining) dermal microvessels (vWF positive; *blue* staining) was significantly higher in bleomycin- vs. saline-treated mice. Vessels are indicated by *arrows*. Pictures are representative examples of four bleomycin-treated and four saline-treated controls. *Ang* Angiopoietin, *HPF* high power field, *vWF* von Willebrand factor, *wt* wildtype

Since inflammation is another characteristic pathogenic feature in early stages of SSc, we next assessed the expression levels of Ang/Tie2 in the model of bleomycin-induced skin fibrosis [28] since it mimics early, inflammatory stages of SSc [29]. Treatment with bleomycin resulted in significant skin fibrosis as measured by increase in dermal thickness (mean ± SEM 1.5 ± 0.05-fold) and hydroxyproline contents (1.6 ± 0.1-fold; *p* < 0.05 each) (Additional file 3). Interestingly, there was a trend towards a higher percentage of Ang-2+ dermal microvessels (vWF+) in bleomycin-challenged mice vs. saline-treated controls (% of Ang-2+/vWF+ microvessels/HPF 95.8 ± 2.7 vs. 65 ± 15; *p* = 0.08) (Fig. 5a). No differences were observed for the expression of Ang-1 in controls and bleomycin-treated mice (% of Ang-1+/vWF+ microvessels/HPF 66.7_(66.7,75)_ vs. 100_(66.7,100)_; *p* = 0.3) (Fig. 5b). The expression of mbTie2 in dermal capillaries (vWF+) was lower in bleomycin-treated mice vs. saline-treated controls (% of Tie2+/vWF+ microvessels/HPF 50_(33,75)_ vs. 100_(94,100)_; *p* = 0.002) (Fig. 5c). These observations are in line with recent data giving evidence of that in inflammatory conditions, the expression of Ang-2 is increased whereas the expression of mbTie2 is decreased [7].

**Fig. 6:**
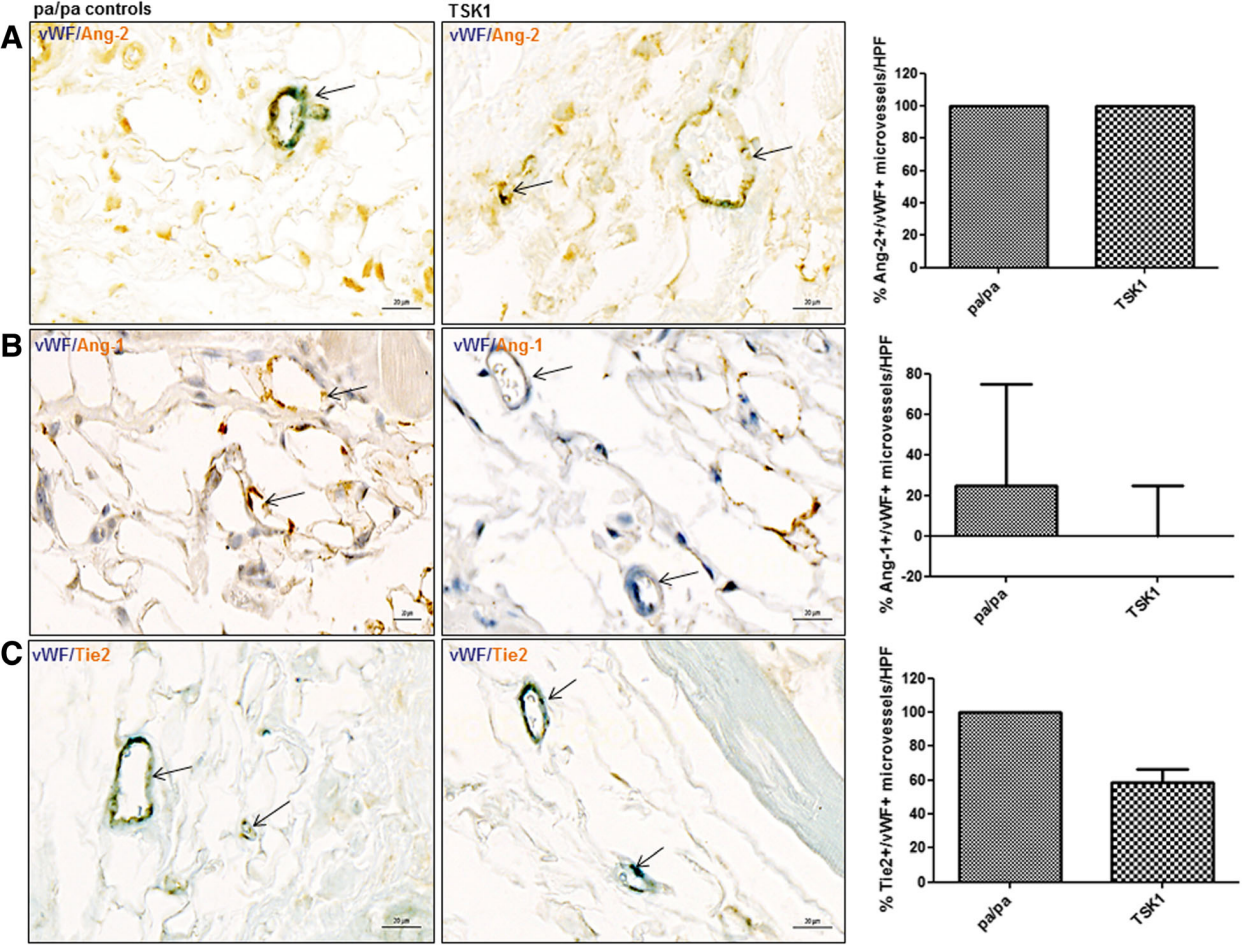
Expression of Ang/Tie2 in dermal microvessels of TSK1 mice. **a**, **b**, **c** In the TSK 1 model, no significant changes could be observed with respect to the expression of Ang-2, Ang-2, Tie2 (*brown* staining) in microvessels (vWF positive; *blue* staining) in the hypodermis. Vessels are indicated by *arrows*. Pictures are representative examples of four TSK mice and four pa/pa controls. *Ang* Angiopoietin, *HPF* high power field, *TSK1* tight skin 1, *vWF* von Willebrand factor

Finally, we investigated the dermal expression of Ang/Tie2 in an inflammation-independent, late-stage model of SSc, the TSK1 model [30]. In TSK1 mice, the expression of Ang-2 in capillaries (vWF+) was not changed compared with pa/pa controls (100_(50,100)_ vs. 100_(88,100)_; *p* = 0.9) (Fig. 6a). No statistically significant difference was observed in TSK1 mice vs. pa/pa controls neither for Ang-1 (% of Ang-1+/vWF+ microvessels/HPF 0_(0,25)_ vs.25_(0,75)_; *p* = 0.2) nor for mbTie2 (% of Tie2+/vWF+ microvessels/HPF 58.4_(13,67)_ vs. 100_(0,100)_, *p* = 0.2) (Fig. 6b, c) although there was a tendency to a reduction of Tie2+ microvessels in TSK1 mice.

## Discussion

In SSc, peripheral microvasculopathy is a key pathogenic event. Our results suggest that a dysbalance of Ang/Tie2 might contribute to the vascular changes. An excess of Ang-2 over Ang-1 – as herein observed on tissue and serum level – is known to induce vascular remodelling by blocking Tie2 signalling on EC [6, 10, 11] (Fig. 1). Interestingly, SSc patients with digital ulcers showed a significantly lower Ang-1/-2 ratio as compared with those without, which further underlines the potential clinical relevance of the observed alterations although the low number of patients and the heterogeneity of the disease warrant some caution in the interpretation of these results. Depending on the absence/presence of VEGF [6, 13], the destabilization of vessels might then lead to regression or (disorganized) sprouting (Fig. 1), both well-described events in SSc microvasculopathy. Moreover, the substantial loss of mbTie2 on dermal microvessels in SSc with concomitant increase in sTie2, which also competes with Ang-1, further impairs Tie2 signalling (Fig. 1). Notably, treatment of dermal HMVECs with VEGF led to a dose-dependent reduction of Tie2+ cells with concomitant increase of sTie2 in the supernatants thereby supporting the shedding hypothesis. Interestingly, a similar dysbalance of Ang-1/-2, sTie2, and VEGF levels was already observed in pre-SSc patients as in patients with established disease indicating that the disturbance of angiogenic signalling networks is an early pathogenic event.

Our data on the expression of angiopoietins and Tie2 in dermal microvessels of SSc patients extend previous, partially conflicting results on the systemic levels of Ang-1/-2 and sTie2 in SSc. Interestingly, all available studies [16, 31–33] as well as our own data found increased systemic levels of Ang-2 without differences regarding lcSSc or dcSSc subtypes [32, 33]. Data on Ang-1 are more ambiguous. Whereas Michalska et al. reported a similar reduction of Ang-1 levels and the Ang-1/Ang-2 ratio in SSc [16], the study by Dunne et al. showed an increase of Ang-1 in the dcSSc subset, but not the combined SSc group as compared with healthy controls [32]. However, apart from discrepancies that might arise from studying such a heterogeneous disease, these differences might also be attributed to the use of plasma [32] instead of serum. Similar to our observations, the serum levels of sTie2 were found to be elevated by Noda et al [17] and Dunne et al. [32], however, the latter study only demonstrating significant differences in the lcSSc subgroup. The study by Gerlicz et al. showed a trend towards higher levels of sTie2 in both SSc subsets compared with healthy controls, yet the data were not statistically significant [33]. But again, all these studies including our own have only analysed very limited numbers of patients. Therefore, the observed changes of serum/plasma angiopoietin and sTie2 levels in SSc and particularly their correlation with clinical (vascular) features warrant a standardized and systematic evaluation in a large, multicentre cohort. Apart from the fact that in most published studies not all three parameters (Ang-1, Ang-2, sTie2) have been simultaneously analysed, some of the current discrepancies of the association with clinical vascular features might arise from differences such as treatment (vasodilating agents, immunosuppressive drugs), the definitions of ulcers and nailfold capillaroscopy patterns used, but also biobank-related issues such as different processing and/or storage of blood samples and use of different ELISA assays. Thus, although the current available data support a dysbalance of the Ang/Tie2 system in SSc, the clinical implications have yet to be elucidated including the performance of more detailed functional in vitro experiments.

Our in vivo data derived from the different animal models, which closely mirrored the changes observed in SSc patients, suggested that VEGF, hypoxia, and inflammation might play a role in the dysbalance of the Ang/Tie2 system in SSc.

## Conclusions

Thus, our data extend the previous concept of peripheral microangiopathy in SSc [1–4, 34] giving novel evidence on tissue level of an even more complex dysregulation of angiogenic key players. The dysregulation of the Ang/Tie-2 system appears to be specific for vascular and inflammatory conditions, as the non-vascular and inflammation-independent TSK1 model did not reflect the changes observed in the human disease. This further underlines that for proof of concept studies the appropriate choice of an animal model that closely reflects the situation of the human disease, is of outmost importance [35]. However, for the development of targeted treatment strategies those interactions and their biologic relevance will first have to be carefully and thoroughly studied in suitable preclinical settings including detailed functional experiments and specific animal models.

## Additional files

Additional file 1: Supplemental methods (DOC 43 kb)

Additional file 2: Figure S1. Induction of skin fibrosis in the murine bleomycin skin model. (A) shows the increase in dermal thickness upon bleomycin treatment (HE staining) whereas (B) depicts the increased deposition of extracellular matrix proteins (Sirius Red staining). (C) and (D) show the semi-quantitative analysis of dermal thickness measurements and of hydroxyproline contents. Pictures are representative examples of four bleomycin-treated and four saline-treated controls. (TIF 71 kb)

Additional file 3: Figure S2. Changes of the expression of angiopoietins and Tie2 in chronic hypoxia in vivo. (A) shows no effect of hypoxia on the levels of Ang-1 mRNA, whereas Ang-2 (B) and Tie2 mRNA transcripts (C) increased compared to normoxia. RNA from three mice kept in hypoxia and nine mice kept in normoxia were analysed by qRT-PCR. (TIF 371 kb)

## Abbreviations

+/-: Heterozygous
+/+: Homozygous
Ang: Angiopoietin
dc: Diffuse cutaneous
EC: Endothelial cells
HMVEC: Human microvascular endothelial cell
HPF: High power field
IHC: Immunohistochemistry
lc: Limited cutaneous
mb: Membrane bound
SEM: Standard error of the mean
SSc: Systemic sclerosis
sTie2: Soluble Tie2
tg: Transgenic
TSK1: Tight skin 1
VEGF: Vascular endothelial growth factor
vWF: von Willebrand factor
wt: Wildtype
