# Supplementary material for: Tie2 as a novel key factor of microangiopathy in systemic sclerosis
Source: Arthritis Res Ther. 2017 May 25;19:105. doi: 10.1186/s13075-017-1304-2 (PMC5445339; doi:10.1186/s13075-017-1304-2)
Supplement: Supplementary file 1 — Supplemental methods (DOC 43 kb) [file 13075_2017_1304_MOESM1_ESM.doc]

**Additional File 1 - Supplemental Methods**

**Histology and analyses of skin sections**

Skin biopsies were fixed in 4% formalin and embedded into paraffin. Five μm thick sections were stained with hematoxylin and eosin (HE) for determination of dermal thickness. Extracellular matrix was stained with picrosirius red according to standard protocols [1].

The analyses of all histologic stainings were performed by two independent examiners who were blinded with regard to the different groups. All slides were analysed twice by each examiner. In case of a variation of the results >10%, the respective slides were re-assessed to reach consensus. Pictures were taken with a digital camera on an Imager1 microscope (Carl-Zeiss AG, Feldbach, Switzerland), using AxioVision software Release 4.6. Dermal thickness was analysed in 3 randomly chosen high power fields per section at x100 magnification by measuring the distance between the epidermal-dermal junction and the dermal-subcutaneous fat junction [1].

**Hydroxyproline assay**

The content of collagens in bleomycin-treated murine skin samples was evaluated by hydroxyproline assay [2]. Briefly, after digestion of punch biopsy specimens (3 mm diameter) in 6*M* HCl for 3 hours at 120°C, the pH of the samples was adjusted to 7 with 6*M* NaOH. Next, samples were mixed with 0.06*M* chloramine T and incubated for 20 minutes at room temperature. Next, 3.15*M* perchloric acid and 20% *p*-dimethylaminobenzaldehyde were added, and samples were incubated for an additional 20 minutes at 60°C. The absorbance was determined at 557 nm with a SpectraMax 190 microplate spectrophotometer (Molecular Devices).

**Supplemental references**

1. Maurer B, Distler A, Dees C, Khan K, Denton CP, Abraham D, Gay RE, Michel BA, Gay S, Hw Distler J *et al*: **Levels of target activation predict antifibrotic responses to tyrosine kinase inhibitors**. *Ann Rheum Dis* 2013, **72**(12):2039-2046.

2. Woessner JF, Jr.: **The determination of hydroxyproline in tissue and protein samples containing small proportions of this imino acid**. *Archives of biochemistry and biophysics* 1961, **93**:440-447.
