# Supplementary figures and images for: Tie2 as a novel key factor of microangiopathy in systemic sclerosis
Source: Arthritis Res Ther. 2017 May 25;19:105. doi: 10.1186/s13075-017-1304-2 (PMC5445339; doi:10.1186/s13075-017-1304-2)

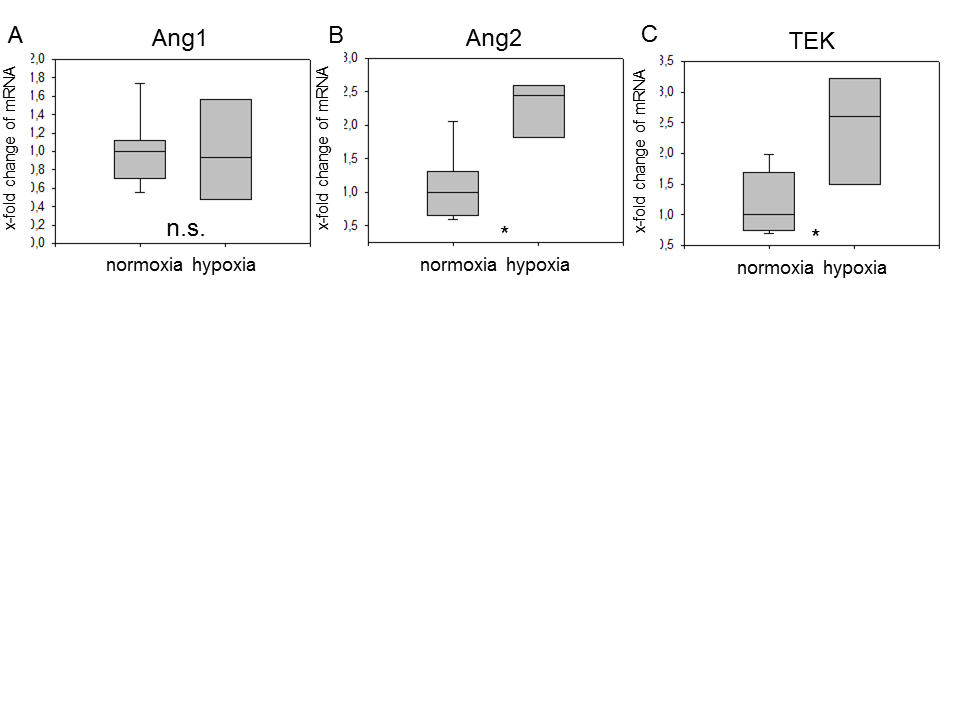

Supplement: Supplementary file 2 — Induction of skin fibrosis in the murine bleomycin skin model. (A) shows the increase in dermal thickness upon bleomycin treatment (HE staining) whereas (B) depicts the increased deposition of extracellular matrix proteins (Sirius Red staining). (C) and (D) show the semi-quantitative analysis of dermal thickness measurements and of hydroxyproline contents. Pictures are representative examples of four bleomycin-treated and four saline-treated controls. (TIF 71 kb) [file 13075_2017_1304_MOESM2_ESM.tif]

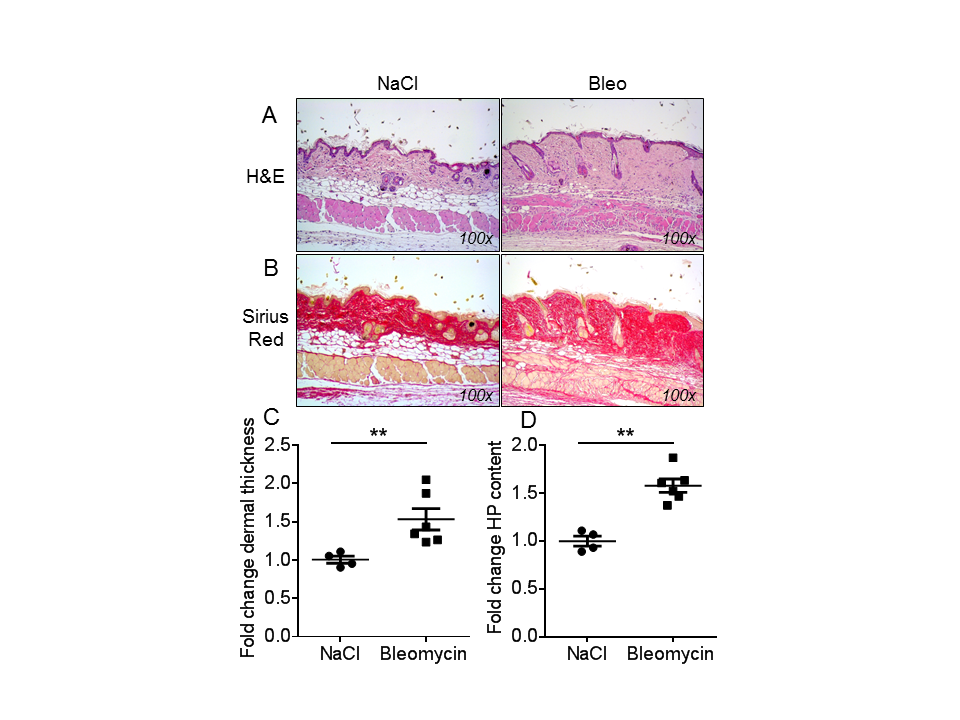

Supplement: Supplementary file 3 — Changes of the expression of angiopoietins and Tie2 in chronic hypoxia in vivo. (A) shows no effect of hypoxia on the levels of Ang-1 mRNA, whereas Ang-2 (B) and Tie2 mRNA transcripts (C) increased compared to normoxia. RNA from three mice kept in hypoxia and nine mice kept in normoxia were analysed by qRT-PCR. (TIF 371 kb) [file 13075_2017_1304_MOESM3_ESM.tif]
